# Global research trends and thematic evolution of respiratory microbiota in COPD: a bibliometric study

**DOI:** 10.3389/fmed.2026.1780141

**Published:** 2026-04-09

**Authors:** Haizong Hu, Mei Yang, Baiwu Liang, Yan Tang, Xiaoli Xie, Lijun Chen, Qixin Wang, Hongxia Yu, Xiaochao Du

**Affiliations:** 1 Department of Respiratory and Critical Care Medicine, Dazhou Hospital of Integrated Traditional Chinese and Western Medicine, Dazhou Second People’s Hospital, Dazhou, China; 2 Department of Oncology, Dazhou Hospital of Integrated Traditional Chinese and Western Medicine, Dazhou Second People's Hospital, Dazhou, China; 3 Department of Laboratory Medicine, Deyang People’s Hospital, Deyang, China

**Keywords:** bibliometric analysis, citation bursts, co-occurrence, COPD, respiratory microbiota

## Abstract

**Background:**

Chronic obstructive pulmonary disease (COPD) is increasingly recognized as a disorder influenced by the respiratory microbiota. Microbial dysbiosis has been linked to disease progression, inflammation, and clinical outcomes. However, a comprehensive overview of the global research landscape and evolving themes in this field is still lacking.

**Methods:**

Publications on COPD and respiratory microbiota were retrieved from the Web of Science Core Collection (WoSCC) and Scopus databases. Bibliometric analyses, including publication trends, co-authorship networks, keyword co-occurrence, citation bursts, and thematic evolution, were conducted using VOSviewer, CiteSpace, and the bibliometrix package in R.

**Results:**

Between 2001 and 2025, 296 publications were identified in WoSCC and 433 in Scopus, reflecting a sustained growth in research output. Keyword co-occurrence and clustering analyses revealed three main research hotspots: (1) respiratory microbiota composition and dynamics, (2) pathogen colonization and inflammation-related processes, and (3) clinically relevant outcomes. Citation burst and thematic evolution analyses demonstrated a clear temporal shift from pathogen-centered studies toward microbiota-based, dynamic, and clinically oriented research paradigms. International collaboration is increasingly prominent, with China, the USA, and the UK leading in productivity and citation impact.

**Conclusion:**

This bibliometric study systematically delineates the intellectual structure and evolving trends of COPD-related respiratory microbiota research. Our findings highlight the maturation of the field, reveal emerging research directions such as multi-omics integration and gut–lung axis interactions, and provide a quantitative reference for guiding future translational and microbiota-focused studies in COPD.

## Introduction

Chronic obstructive pulmonary disease (COPD) is a common and progressive respiratory disorder characterized by persistent airflow limitation, chronic inflammation, and structural changes in the lung that result in significant morbidity and mortality worldwide. Despite advances in treatment, COPD remains among the leading causes of death globally, and current therapeutic strategies mainly target symptomatic relief rather than underlying disease mechanisms (1, 2). Traditionally, COPD pathogenesis has emphasized host immune responses to noxious exposures such as smoking and air pollution; however, these factors alone do not fully explain the heterogeneous clinical phenotypes and disease progression observed among patients (3).

Over the past decade, the advent of next-generation sequencing and metagenomic analyses has shifted the paradigm regarding the lower respiratory tract, revealing that healthy lungs are not sterile but instead harbor a diverse microbial community—the respiratory microbiota (4, 5). These microbial communities, including bacteria, fungi, and viruses, interact with host immune responses and contribute to mucosal homeostasis under physiological conditions (4, 6). Studies employing high-throughput sequencing have shown that the structure and diversity of the respiratory microbiota differ substantially between healthy individuals and COPD patients, with COPD associated with reduced microbial diversity and an increased prevalence of potential pathogens such as *Haemophilus*, *Moraxella*, and *Pseudomonas* (7, 8). Alterations in respiratory microbial communities have also been linked with COPD severity, inflammatory profiles, and the frequency of exacerbations, supporting a role for microbiota–host interactions in disease progression (9, 10).

Despite these advances, the mechanistic underpinnings of how altered respiratory microbiota contribute to COPD remain incompletely understood. Recent multi-omics studies indicate that microbial dysbiosis may influence host immunity, metabolic pathways, and epithelial barrier function, potentially exacerbating chronic inflammation and tissue damage in COPD (11). For example, integrated metagenomic and transcriptomic analyses have revealed distinct microbial signatures associated with severe airflow limitation and elevated pro-inflammatory pathways, suggesting that microbiota composition may modulate disease severity and patient outcomes (11).

Beyond local respiratory communities, emerging evidence also supports the existence of a gut–lung axis, wherein bidirectional communication between the intestinal microbiota and respiratory tract influences immune homeostasis and disease susceptibility (12, 13). Clinical and experimental studies report that COPD patients exhibit gut microbiota dysbiosis, characterized by altered microbial diversity and metabolic profiles, which may contribute to systemic inflammation and pulmonary dysfunction (12, 14). Animal models further demonstrate that gut microbial changes can modulate lung immune responses and exacerbate COPD-like pathology, implicating gut microbiota as a potential modifier of respiratory disease (15).

Collectively, these findings point to an intricate interplay among respiratory microbiota, systemic microbial communities, and host immune responses in COPD pathogenesis. However, research in this area has expanded rapidly and remains highly heterogeneous in terms of study design, analytical methods, and focal themes (16). A comprehensive, quantitative synthesis of global research activity—including publication trends, collaborative networks, and thematic evolution—is therefore needed to clarify the intellectual landscape and identify emerging hotspots in COPD microbial research.

In this study, we performed a bibliometric analysis of publications on the respiratory microbiota in COPD indexed in the Web of Science Core Collection (WoSCC) and Scopus databases. Unlike traditional meta-analyses that focus on specific microbial indices, this bibliometric study aims to map the intellectual landscape, identify emerging trends, and clarify research priorities in COPD-related respiratory microbiota research. By integrating co-authorship networks, keyword co-occurrence patterns, citation bursts, and thematic evolution, we systematically mapped the development, knowledge structure, and evolving trends in this rapidly growing research field.

## Methods

This study adopted a bibliometric and visualized analysis to systematically evaluate research on the respiratory microbiota in COPD. Publications related to the respiratory microbiota and COPD were retrieved from the WoSCC and Scopus databases. The retrieval time span was from 2000 to 2025, and all records were downloaded on December 18, 2025 to avoid database update bias. Only original articles and review articles published in English were included.

### Data source and study design

This study employed bibliometric and visualization analyses to systematically evaluate research focusing on the respiratory microbiota in COPD. Publications related to the respiratory microbiota and COPD were retrieved from the WoSCC and Scopus databases. The retrieval time span covered the period from 2000 to 2025, and all records were downloaded on December 18, 2025, to avoid bias caused by database updates. Only original research articles and review articles published in English were included in this study.

### Search strategy

The search strategy combined disease-related terms (“COPD” OR “chronic obstructive pulmonary disease”) with microbiota-related terms (“respiratory microbiota,” “respiratory microbiome,” and their related variants). The complete and detailed search strategies for each database are provided in the Supplementary materials.

### Data extraction and cleaning

All retrieved records were exported with complete bibliographic information. WoSCC records were downloaded in plain text format with full records and cited references, whereas Scopus records were exported in CSV format with complete author, affiliation, and keyword information. A detailed screening process was conducted. Duplicate records were identified and removed using the bibliometrix package in R and manual verification. Titles and abstracts were screened independently to ensure relevance. In addition, a PRISMA-style flow diagram has been added as Supplementary Figure S1 to visually demonstrate identification, screening, eligibility, and final inclusion. Data cleaning was performed before analysis, including author name disambiguation, institutional name normalization, and keyword unification. Synonymous keywords (e.g., “respiratory microbiota” and “lung microbiome”) were merged to ensure analytical consistency. The data-cleaning process was independently conducted by two researchers, and any discrepancies were resolved through discussion.

### Bibliometric analysis using bibliometrix

Descriptive bibliometric analysis was conducted using the bibliometrix package in R software (version 4.5.1). Data from WoSCC and Scopus were imported using the convert2df () function with database-specific parameters. Core bibliometric indicators—including annual scientific production, document types, source journals, authorship patterns, country contributions, and citation metrics—were obtained using the biblioAnalysis () function. Visualization of bibliometric indicators was performed using the built-in plotting functions of Bibliometrix.

### Network analysis using VOSviewer

Network visualization and mapping analyses were performed using VOSviewer based on data from WoSCC and Scopus. Co-authorship (authors and countries), keyword co-occurrence, and source citation networks were constructed using the full counting method. Thresholds for inclusion were set according to network size to ensure interpretability. In the WoSCC database, minimum thresholds were set at ≥4 publications for countries, institutions, and authors in co-authorship analyses. For co-citation analysis, the minimum citation counts were ≥40 for source journals, ≥10 for cited references, and ≥15 for cited authors. For keyword co-occurrence analysis, the minimum frequency threshold was ≥5. In the Scopus database, minimum thresholds for co-authorship analysis were ≥3 publications for countries and ≥4 publications for institutions and authors. For co-citation analysis in Scopus, the minimum citation counts were ≥10 for source journals, ≥5 for cited references, and ≥10 for cited authors. For keyword co-occurrence analysis, the minimum frequency threshold was ≥20.

### Keyword burst detection using CiteSpace

To identify research hotspots, keyword burst detection was performed using CiteSpace based on the WoSCC dataset. Citation burst detection was applied to identify keywords with rapidly increasing citation frequencies, representing emerging research fronts.

## Results

### General characteristics of the publications

Based on the WoSCC, a total of 296 publications related to the respiratory microbiota in COPD were identified between 2001 and 2025, spanning 128 journals. These publications comprised 209 original articles, 85 review articles, and 2 early-access articles (Supplementary Table S1). The average number of citations per document was 48.22, with an average of 4.49 citations per year per document. The overall annual growth rate of publications was 14.72%, reflecting a sustained increase in research output over time.

433 publications were published between 2005 and 2025 in the Scopus database, covering 185 source journals. These included 276 original articles and 157 review articles (Supplementary Table S1). The average citations per document and the average citations per year per document were 48.8 and 4.37, respectively. The annual growth rate of publications was 16.44%, indicating a comparable and slightly higher expansion rate than that observed in the WoSCC dataset.

### Annual scientific production

**Figure 1:**
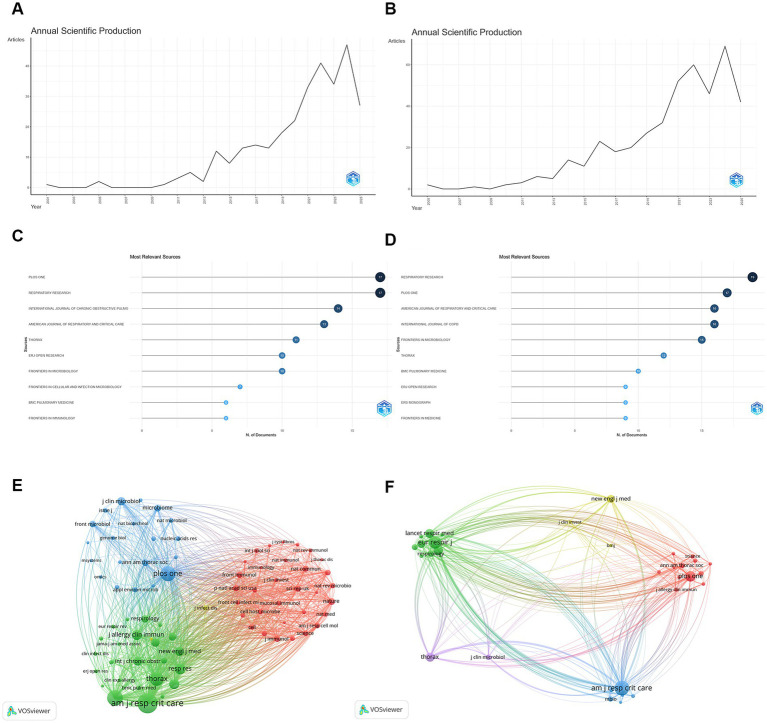
Bibliometric characteristics of publications on respiratory microbiota in COPD. **(A)** Annual scientific production based on the WoSCC database. **(B)** Annual scientific production based on the Scopus database. **(C)** Distribution of the most productive journals identified from the WoSCC database. **(D)** Distribution of the most productive journals identified from the Scopus database. **(E)** Journal co-citation network constructed using WoSCC data. **(F)** Journal co-citation network constructed using Scopus data. Node size represents citation frequency, and links indicate co-citation relationships between journals.

According to the WoSCC data, annual scientific production exhibited a clear upward trend (Figure 1A). Before 2013, fewer than five publications were produced annually. From 2014 onward, publication output increased steadily, reaching a peak of 47 publications in 2024, followed by 27 publications in 2025 (partial year). A similar pattern was observed in the Scopus-based analysis (Figure 1B). Publication output remained relatively low before 2013, followed by a marked increase after 2014, with a maximum of 69 publications recorded in 2024.

### Distribution of journals and core sources

Source analysis based on the WoSCC database showed that studies on the respiratory microbiota in COPD were primarily published in journals related to respiratory medicine and microbiology. PLoS One [impact factor (IF) = 2.6] and Respiratory Research (IF = 5.0) were the most productive journals, each publishing 17 articles. They were followed by the International Journal of Chronic Obstructive Pulmonary Disease (*n* = 14, IF = 3.1), American Journal of Respiratory and Critical Care Medicine (*n* = 13, IF = 19.4), and Thorax (*n* = 11, IF = 7.7) (Figure 1C; Table 1).

**Table 1:** Top 10 journals with the most published articles.

Sources	Articles	IF (2024)
WoSCC		
PLoS One	17	2.6
Respiratory Research	17	5
International Journal of Chronic Obstructive Pulmonary Disease	14	3.1
American Journal of Respiratory and Critical Care Medicine	13	19.4
Thorax	11	7.7
ERJ Open Research	10	4
Frontiers in Microbiology	10	4.5
Frontiers in Cellular and Infection Microbiology	7	4.8
BMC Pulmonary Medicine	6	2.8
Frontiers in Immunology	6	5.9
Scopus		
Respiratory Research	19	5
PLoS One	17	2.6
American Journal of Respiratory and Critical Care Medicine	16	19.4
International Journal of Chronic Obstructive Pulmonary Disease	16	3.1
Frontiers in Microbiology	15	4.5
Thorax	12	7.7
BMC Pulmonary Medicine	10	2.8
ERJ Open Research	9	4
ERS Monograph	9	NA
Frontiers in Medicine	9	3

Citation analysis further identified the most frequently cited journals (Table 2). American Journal of Respiratory and Critical Care Medicine ranked first with 1,726 citations, followed by European Respiratory Journal (1,038 citations), PLoS One (958 citations), Thorax (791 citations), and Respiratory Research (491 citations). Scopus-based source analysis demonstrated a similar journal distribution pattern. The most productive journals included Respiratory Research (*n* = 19, IF = 5.0), PLoS One (*n* = 17, IF = 2.6), American Journal of Respiratory and Critical Care Medicine (*n* = 16, IF = 19.4), and the International Journal of Chronic Obstructive Pulmonary Disease (*n* = 16, IF = 3.1) (Figure 1D; Table 1). These journals largely overlapped with the core sources identified in the WoSCC analysis.

**Table 2:** Top 10 journals with the most cited journals (WoSCC).

WoSCC		
Sources	Cites	IF (2024)
American Journal of Respiratory and Critical Care Medicine	1726	19.4
European Respiratory Journal	1,038	21.0
PLoS One	958	2.6
Thorax	791	7.7
Respiratory Research	491	5.0
Chest	434	8.6
Journal of Allergy and Clinical Immunology	434	11.4
The New England Journal of Medicine	336	78.5
International Journal of Chronic Obstructive Pulmonary Disease	325	3.1
Lancet	309	88.5

Co-citation analyses based on both WoSCC and Scopus data (Figures 1E,F) consistently identified American Journal of Respiratory and Critical Care Medicine as the central hub of shared references, indicating its prominent position within the citation network of COPD and respiratory microbiota research.

### Most cited references and citation bursts

Using the Bibliometrix package in R, the top 10 most cited references related to COPD and the respiratory microbiota were identified in both databases (Table 3). In the WoSCC dataset, each of these articles had received more than 250 citations and was distributed across eight journals. Representative highly cited publications included “The immune response to *Prevotella* bacteria in chronic inflammatory disease,” “Analysis of the lung microbiome in the ‘healthy’ smoker and in COPD,” and “The lung tissue microbiome in chronic obstructive pulmonary disease.”

**Table 3:** Top 10 cited references related to the relationship between COPD and respiratory microbiota.

Paper	Title	Total citations
WoSCC		
Larsen JM, 2017, Immunology	The immune response to *Prevotella* bacteria in chronic inflammatory disease	1,002
Erb-Downward JR, 2011, PLoS One	Analysis of the lung microbiome in the “healthy” smoker and in COPD	731
Sze Ma, 2012, Am J Resp Crit Care	The lung tissue microbiome in chronic obstructive pulmonary disease	423
Dickson RP, 2014, Lancet	The role of the microbiome in exacerbations of chronic lung diseases	366
Pragman AA, 2012, PLoS One	The lung microbiome in moderate and severe chronic obstructive pulmonary disease	328
Budden KF, 2019, Lancet Resp Med	Functional effects of the microbiota in chronic respiratory disease	319
Wang Z, 2016, Eur Respir J	Lung microbiome dynamics in COPD exacerbations	304
Molyneaux PL, 2013, Am J Resp Crit Care Med	Outgrowth of the bacterial respiratory microbiota after rhinovirus exacerbation of chronic obstructive pulmonary disease	296
Bowerman KL, 2020, Nat Commun	Disease-associated gut microbiome and metabolome changes in patients with chronic obstructive pulmonary disease	287
Beck JM, 2012, Transl Res	The microbiome of the lung	273
Scopus		
Hilty M, 2010, PLoS One	Disordered microbial communities in asthmatic airways	1,510
Larsen JM, 2017, Immunology	The immune response to *Prevotella* bacteria in chronic inflammatory disease	1,077
Erb-Downward JR, 2011, PLoS One	Analysis of the lung microbiome in the “healthy” smoker and in COPD	806
Sze Ma, 2012, Am J Resp Crit Care	The lung tissue microbiome in chronic obstructive pulmonary disease	476
Pragman AA, 2012, PLoS One	The lung microbiome in moderate and severe chronic obstructive pulmonary disease	372
Budden KF, 2019, Lancet Resp Med	Functional effects of the microbiota in chronic respiratory disease	346
Wang Z, 2016, Eur Respir J	Lung microbiome dynamics in COPD exacerbations	346
Molyneaux PL, 2013, Am J Resp Crit Care Med	Outgrowth of the bacterial respiratory microbiota after rhinovirus exacerbation of chronic obstructive pulmonary disease	340
Beck JM, 2012, Transl Res	The microbiome of the lung	316
Bowerman KL, 2020, Nat Commun	Disease-associated gut microbiome and metabolome changes in patients with chronic obstructive pulmonary disease	315

To further identify emerging research fronts, CiteSpace was applied to detect the top 25 references with the strongest citation bursts in the WoSCC dataset (Table 4; Supplementary Table S2). The three strongest citation bursts were associated with “Lung microbiome dynamics in COPD exacerbations” (strength = 20.71), “The lung tissue microbiome in chronic obstructive pulmonary disease” (strength = 15.31), and “Respiratory microbiota dynamics in exacerbations of chronic obstructive pulmonary disease” (strength = 14.74).

**Table 4:** Top 25 references with the strongest citation bursts in COPD and respiratory microbiota based on WoSCC.

References	Burst	Burst begin	Burst end
Wang Z, 2016, Eur Respir J, V47, P1082	20.71	2017	2021
Sze MA, 2012, Am J Resp Crit Care, V185, P1073	15.31	2013	2017
Huang YJ, 2014, J Clin Microbiol, V52, P2813	14.74	2015	2019
Erb-Downward JR, 2011, PLoS One, V6, P0	13.67	2012	2016
Mayhew D, 2018, Thorax, V73, P422	12.58	2019	2022
Hilty M, 2010, PLoS One, V5, P0	12.16	2011	2015
Molyneaux PL, 2013, Am J Resp Crit Care, V188, P1224	12.01	2014	2018
Huang YJ, 2010, Omics, V14, P9	11.58	2011	2015
Garcia-Nuñez M, 2014, J Clin Microbiol, V52, P4217	11.04	2016	2019
Pragman AA, 2012, PLoS One, V7, P0	10.84	2014	2017
Ramsheh MY, 2021, Lancet Microbe, V2, PE300	10.32	2023	2025
Wang Z, 2021, Am J Resp Crit Care, V203, P1488	10.23	2022	2025
Wang Z, 2018, Thorax, V73, P331	10.02	2019	2023
Millares L, 2014, Eur J Clin Microbiol, V33, P1101	9.89	2015	2019
Charlson ES, 2011, Am J Resp Crit Care, V184, P957	9.24	2012	2016
Morris A, 2013, Am J Resp Crit Care, V187, P1067	9.09	2014	2018
Sze MA, 2015, Am J Resp Crit Care, V192, P438	8.97	2017	2020
Dicker AJ, 2021, J Allergy Clin Immun, V147, P158	8.65	2022	2025
Cabrera-Rubio R, 2012, J Clin Microbiol, V50, P3562	8.23	2014	2017
Einarsson GG, 2016, Thorax, V71, P795	8.01	2018	2021
Yan ZZ, 2022, Nat Microbiol, V7, P1361	7.53	2023	2025
Budden KF, 2019, Lancet Resp Med, V7, P907	7.46	2022	2023
Bowerman KL, 2020, Nat Commun, V11, P0	6.83	2022	2025
Leung JM, 2017, Respirology, V22, P634	6.81	2020	2022
Han MK, 2012, Thorax, V67, P456	6.74	2012	2017

The most recent citation bursts were linked to studies focusing on respiratory microbiota composition, inflammatory endotypes, longitudinal multicohort analyses, sputum microbiota profiles, mortality, and multi-omics approaches. These burst references were characterized by cohort-based designs or systematic reviews and exhibited concentrated citation activity over relatively short time periods.

### Authors and collaboration patterns

**Figure 2:**
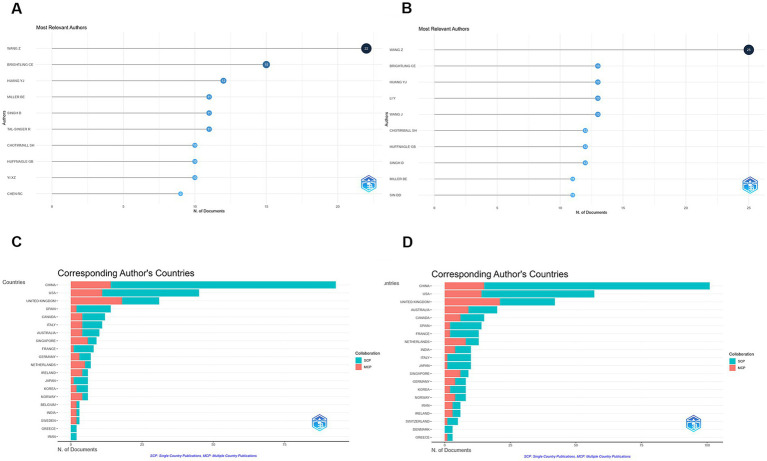
Author productivity and country-level contributions in COPD and respiratory microbiota research. **(A)** Most productive authors based on publication counts in the WoSCC database. **(B)** Most productive authors based on publication counts in the Scopus database. **(C)** Country-level publication output derived from the WoSCC database. **(D)** Country-level publication output derived from the Scopus database.

In the WoSCC dataset, a total of 1,862 authors contributed to publications in this field, with an average of 9.03 authors per document. Only seven single-authored publications were identified, and 31.08% of the publications involved international collaboration. Wang Z was the most productive author with 22 publications, followed by Brightling CE (15 publications) and Huang YJ (12 publications) (Figure 2A).

In the Scopus dataset, 2,312 authors were involved, with an average of 8.43 authors per document. Single-authored publications accounted for 11 documents, and internationally co-authored publications represented 30.02% of the total. The most productive authors included Wang Z (25 publications), Brightling CE (13 publications), and Huang YJ (13 publications) (Figure 2B).

### Country collaboration and citation impact

Country-level analysis based on WoSCC data showed that China was the most productive country with 93 publications, followed by the United States (45 publications) and the United Kingdom (31 publications) (Figure 2C). Publications from the United Kingdom exhibited the highest proportion of international collaboration (Table 5). In terms of citation impact, the United States ranked first with 3,989 total citations, followed by the United Kingdom (2,135 citations) and China (2,134 citations) (Table 6).

**Table 5:** Most relevant countries by corresponding authors.

Country	Articles	Articles %	SCP	MCP	MCP %
WoSCC					
China	93	31.4	79	14	15.1
USA	45	15.2	34	11	24.4
UK	31	10.5	13	18	58.1
Spain	14	4.7	12	2	14.3
Canada	12	4.1	8	4	33.3
Italy	11	3.7	7	4	36.4
Australia	10	3.4	6	4	40
Singapore	9	3	3	6	66.7
France	8	2.7	7	1	12.5
Germany	7	2.4	4	3	42.9
Scopus					
China	101	23.3	86	15	14.9
USA	57	13.2	43	14	24.6
UK	42	9.7	21	21	50
Australia	20	4.6	11	9	45
Canada	15	3.5	9	6	40
Spain	14	3.2	12	2	14.3
France	13	3	11	2	15.4
Netherlands	13	3	5	8	61.5
India	10	2.3	6	4	40
Italy	10	2.3	9	1	10

**Table 6:** Most cited countries of the relationship between COPD and respiratory microbiota.

Country	Citations	Average article citations
WoSCC		
USA	3,989	88.6
UK	2,135	68.9
China	2,134	22.9
Australia	1,038	103.8
Canada	1,002	83.5
Denmark	1,002	1,002.00
Spain	643	45.9
Singapore	489	54.3
Ireland	275	45.8
Italy	230	20.9
Scopus		
USA	4,597	80.6
UK	2,585	61.5
China	2,412	23.9
Australia	1,443	72.2
Denmark	1,294	431.3
Canada	1,059	70.6
Spain	679	48.5
France	576	44.3
Singapore	443	49.2
Netherlands	406	31.2

Similar patterns were observed in the Scopus database (Figure 2D). China, the United States, and the United Kingdom led in publication volume. The Netherlands exhibited the highest proportion of internationally co-authored publications (61.5%) (Table 5). The United States ranked first in total citations (4,597), followed by the United Kingdom (2,585) and China (2,412) (Table 6).

### Co-authorship and collaboration networks

**Figure 3:**
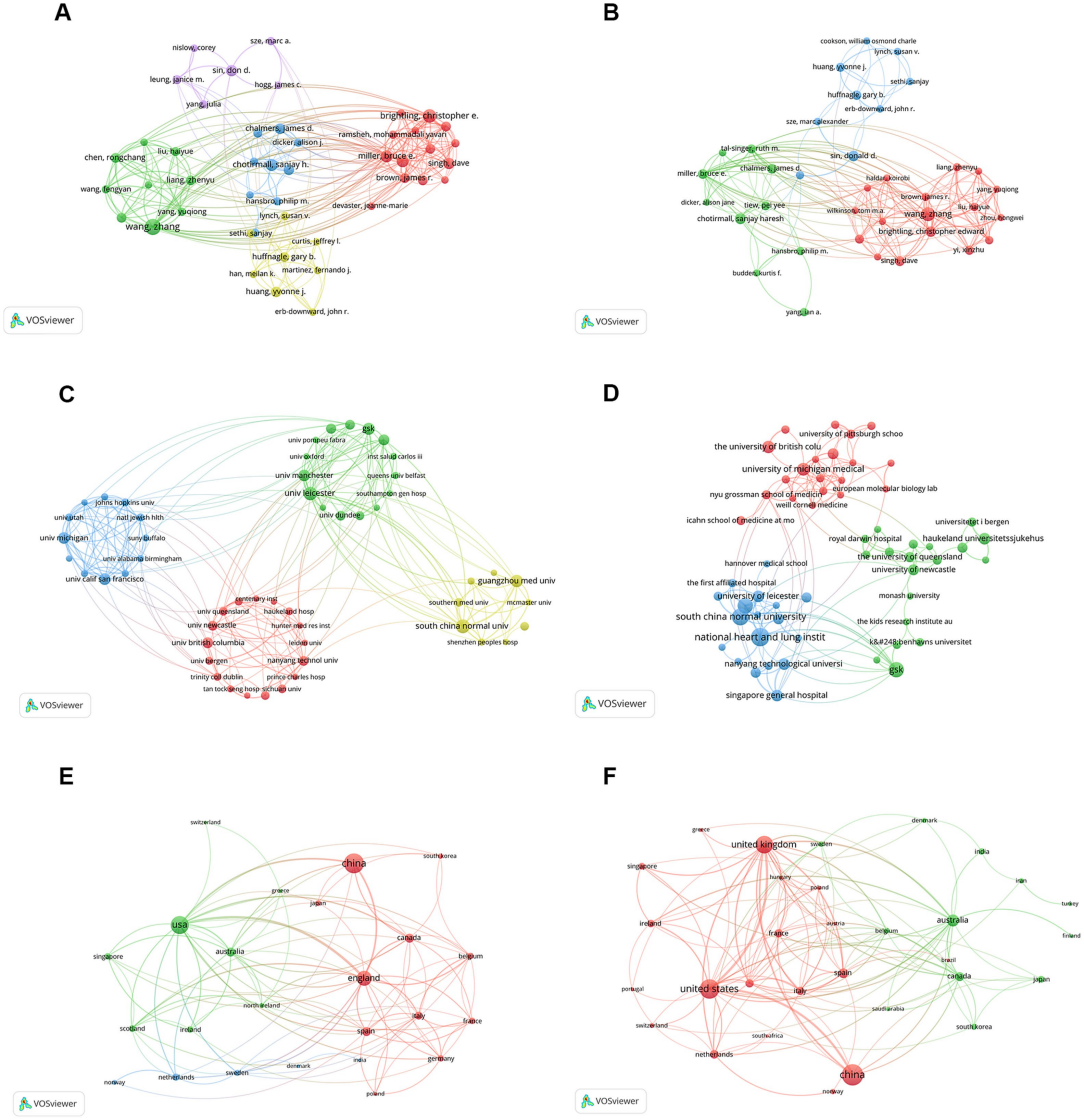
Network analysis of research outputs in COPD and respiratory microbiota studies. **(A)** Co-authorship network analysis based on the WoSCC database. **(B)** Co-authorship network analysis based on the Scopus database. **(C)** Inter-institutional collaboration network derived from the WoSCC database. **(D)** Inter-institutional collaboration network derived from the Scopus database. **(E)** Country-level collaboration network based on the WoSCC database. **(F)** Country-level collaboration network based on the Scopus database. In all networks, node size corresponds to publication volume, and links represent collaborative relationships.

VOSviewer-based co-authorship network analysis of WoSCC data revealed a dense collaboration structure with multiple well-defined author clusters centered around leading researchers (Figure 3A; Table 7). Institutional collaboration networks further indicated extensive cooperation among major research centers. Comparable clustering patterns were observed in the Scopus-based co-authorship and institutional collaboration networks, although differences in network density and link strength were evident (Figure 3B; Table 7).

**Table 7:** Most relevant authors of the relationship between COPD and respiratory microbiota.

Author	Articles	Country	Affiliation
WoSCC			
Wang, Zhang	22	China	South China Normal University
Brightling, Christopher E.	15	UK	University of Leicester
Huang, Yvonne J.	12	USA	University of Michigan System
MILLER BE	11	USA	GlaxoSmithKline
Singh, Dave	11	UK	University of Manchester
Ruth Tal-Singer	11	USA	GlaxoSmithKline
Chotirmall, Sanjay H.	10	Singapore	Singapore General Hospital
Huffnagle, Gary B.	10	USA	University of Michigan System
Yi, Xinzhu	10	China	South China Normal University
Chen, Rongchang	9	China	South China Normal University
Scopus			
Wang, Zhang	25	China	South China Normal University
Brightling, Christopher E.	13	UK	University of Leicester
Huang, Yvonne J.	13	USA	University of Michigan System
Li, Yao	13	China	Xuzhou Medical University
Wang, Jianwei	13	China	Chinese Academy of Medical Sciences & Peking Union Medical College
Chotirmall, Sanjay H.	12	Singapore	Singapore General Hospital
Huffnagle, Gary B.	12	USA	University of Michigan System
Singh, Dave	12	UK	University of Manchester
Miller, Bruce E.	11	USA	GlaxoSmithKline
Sin, Don D.	11	Canada	St. Paul’s Hospital

Institutional collaboration analysis demonstrated that institutions were grouped into several distinct clusters, primarily centered on universities, hospitals, and research institutes specializing in respiratory medicine and microbiology (Figures 3C,D; Table 8). Across both databases, GlaxoSmithKline and the University of British Columbia appeared as major collaboration hubs.

**Table 8:** Most relevant affiliations of the relationship between COPD and respiratory microbiota.

Affiliation	Articles
WoSCC	
Glaxosmithkline	56
University of Michigan	35
University of Michigan System	35
University of British Columbia	33
Astrazeneca	31
University of Leicester	31
University of California System	30
University of Southampton	30
US Department of Veterans Affairs	25
Veterans Health Administration (VHA)	24
University of Minnesota System	23
University of Minnesota Twin Cities	23
Imperial College London	21
South China Normal University	20
University of California San Francisco	19
GuangZhou Medical University	17
Institut National De La Sante Et De La Recherche Medicale (INSERM)	17
ST. PAUL’S Hospital	17
University of Dundee	17
University of Bergen	16
Scopus	
University of British Columbia	28
University of Michigan Medical School	25
National Heart and Lung Institute	23
South China Normal University	21
The First Affiliated Hospital of GuangZhou Medical University	18
Southern Medical University	16
University of Leicester	16
National TaiWan University Hospital	14
UCSF School of Medicine	14
University of Minnesota Twin Cities	14
West China School of Medicine/West China Hospital of SiChuan University	14
University of California	13
University of Southampton	13
Centro De InvestigaciÓn BiomÉdica En Red De Enfermedadea Respiratorias	12
Haukeland Universitetssjukehus	12
Nyu Grossman School of Medicine	12
University of Technology Sydney	12
University of Pittsburgh School of Medicine	11
The University of Queensland	10
TongJi University	10

Country-level collaboration networks based on WoSCC and Scopus data revealed extensive international cooperation, with China, the United States, and the United Kingdom occupying central positions and exhibiting strong collaborative links with multiple countries (Figures 3E,F).

**Figure 4:**
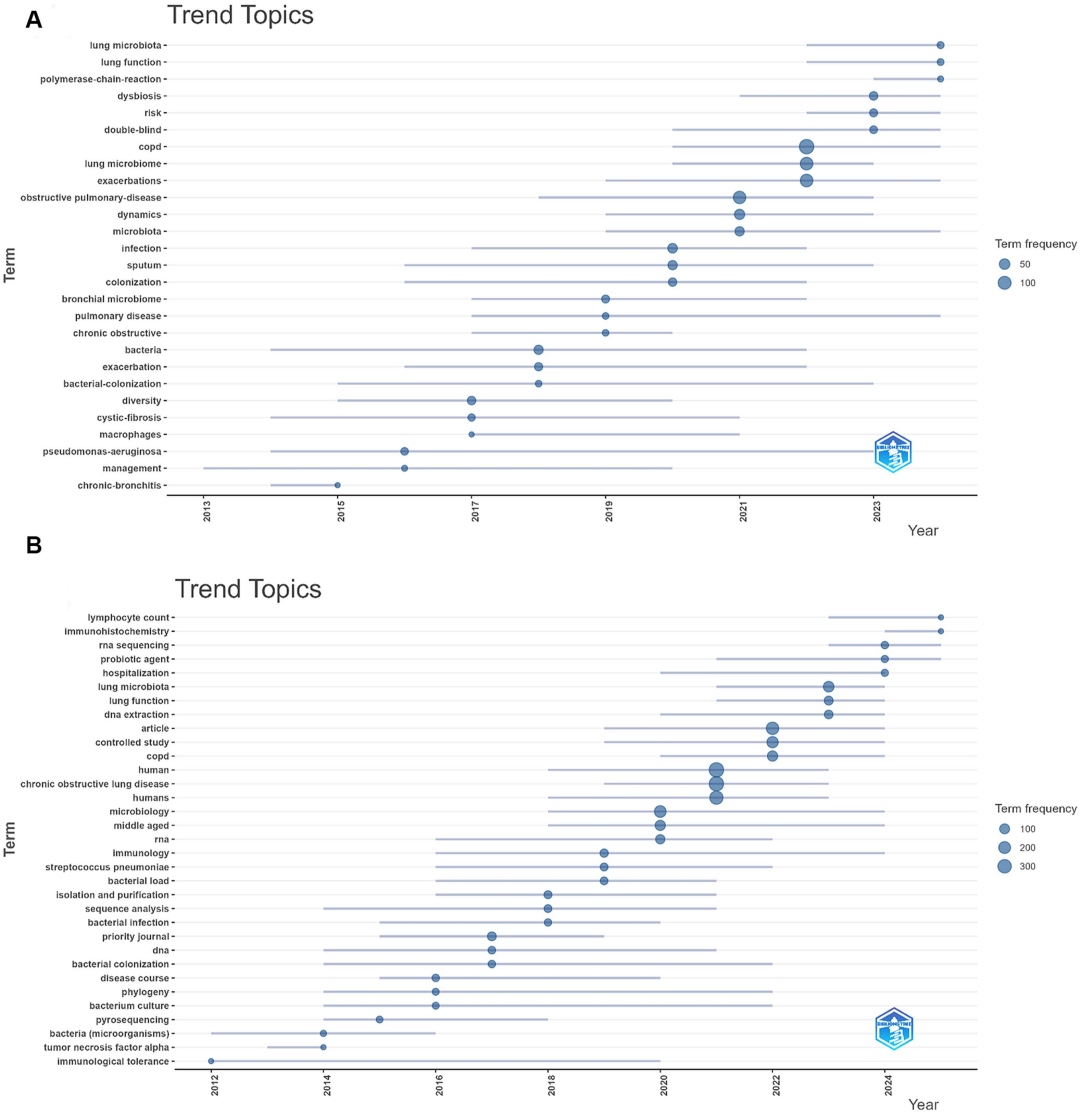
Keyword co-occurrence analysis of publications related to COPD and respiratory microbiota. **(A)** Keyword co-occurrence network based on the WoSCC database. **(B)** Keyword co-occurrence network based on the Scopus database. Nodes represent keywords, with node size indicating frequency of occurrence. Colors denote different keyword clusters identified based on co-occurrence patterns.

Keyword co-occurrence and thematic clusters Keyword co-occurrence analysis using VOSviewer identified 1,143 keywords in the WoSCC dataset of which 60 met the minimum occurrence threshold (≥5 times). The most frequent keywords included inflammation (*n* = 86) infection (*n* = 52) dynamics (*n* = 45) and pathogenesis (*n* = 31) (Table 9). These keywords were grouped into four distinct clusters based on their co-occurrence patterns (Figure 4A).

**Table 9:** Top 20 keywords related to the relationship between COPD and respiratory microbiota.

Words	Occurrences
WoSCC	
Inflammation	86
Infection	52
Dynamics	45
Pathogenesis	31
Gene expression	23
Gut microbiota	23
Cystic fibrosis	22
Diversity	22
Dysbiosis	22
Colonization	21
Lung function	20
Clinical trial	17
Risk	17
Smokers	17
Bronchiectasis	16
Severity	14
Gut–lung axis	13
Biomarker	11
Emphysema	11
Inhaled corticosteroids	11
Scopus	
Disease exacerbation	143
Lung function	132
Genetics	105
Inflammation	105
Microbial diversity	99
Immunology	96
Microbial community	95
Disease severity	79
Inhaled corticosteroids	79
Dysbiosis	75
Animal experiment	73
Antibiotic agent	68
Gut microbiota	62
Bronchoalveolar lavage fluid	59
Disease progression	58
Pneumonia	57
Smokers	57
DNA extraction	56
Pathogenesis	56
Metagenomics	55

Cluster 1 (green) was centered on respiratory inflammation and biomarkers. Representative terms included eosinophils, biomarker, gene expression, together with intervention- and outcome-related keywords (inhaled corticosteroids, mortality, and phenotypes), highlighting its translational and clinical orientation.

Cluster 2 (yellow) emphasized the gut–lung axis, metabolic pathways, and immune regulation. Keywords such as gut microbiota, gut–lung axis, chain fatty acids, immunity, dietary fiber, reflected emerging interdisciplinary research directions.

Cluster 3 (blue) focused on respiratory infection, and inflammatory mechanisms. Keywords such as infection, dynamics, communities, exposure, macrophages, metagenomics, pathogenesis, quality-of-life, risk-factors, and short-term response characterized a mechanism-oriented cluster addressing pathogen-driven respiratory inflammation and host–microbe interactions in COPD.

Cluster 4 (red) represented a COPD-centered core cluster emphasizing overall disease characteristics, clinical outcomes, and microbial diversity. This cluster included keywords related to lung function, symptoms, disease severity, and bronchoalveolar lavage, together with microbial diversity–related terms such as colonization, frequency, prevalence, and severity. Management- and outcome-related terms (management, and pneumonia) further reflected the clinical relevance of this cluster.

All keywords and their frequencies are detailed in Supplementary Table S3.

From the Scopus database, 3,982 keywords were extracted using VOSviewer, of which 71 met the minimum occurrence threshold (≥20 times) and were included in the analysis. Table 9 lists the top 20 most frequent terms, with disease exacerbation (*n* = 143) as the most common, followed by lung function (*n* = 132), genetics (*n* = 105), inflammation (*n* = 105), and microbial diversity (*n* = 99). Cluster analysis revealed three major groups (Figure 4B).

The blue cluster highlighted disease, and the related keywords include animal experiment, pneumonia, lung cancer, pathogenesis, and probiotic agent.

The red cluster focused on acute exacerbations, pathogen infection, and clinical outcomes, characterized by terms including disease exacerbation, disease severity, prevalence, hospitalization, complication, risk factor, disease course, emphysema, antibiotic agent, and disease progression.

The green cluster emphasized respiratory microbiota structure, sequencing approaches, and population-based characteristics, featuring keywords such as microbial diversity, microbial community, bronchoalveolar lavage fluid, DNA extraction, metagenomics, and clinical feature.

The yellow cluster highlighted inflammatory and immune mechanisms and host responses, with keywords related to inflammation, biomarker, eosinophil, interleukin 8, interleukin 6, and interleukin 1beta.

All keyword data are provided in Supplementary Table S3.

### Keyword bursts and thematic evolution

**Figure 5:**
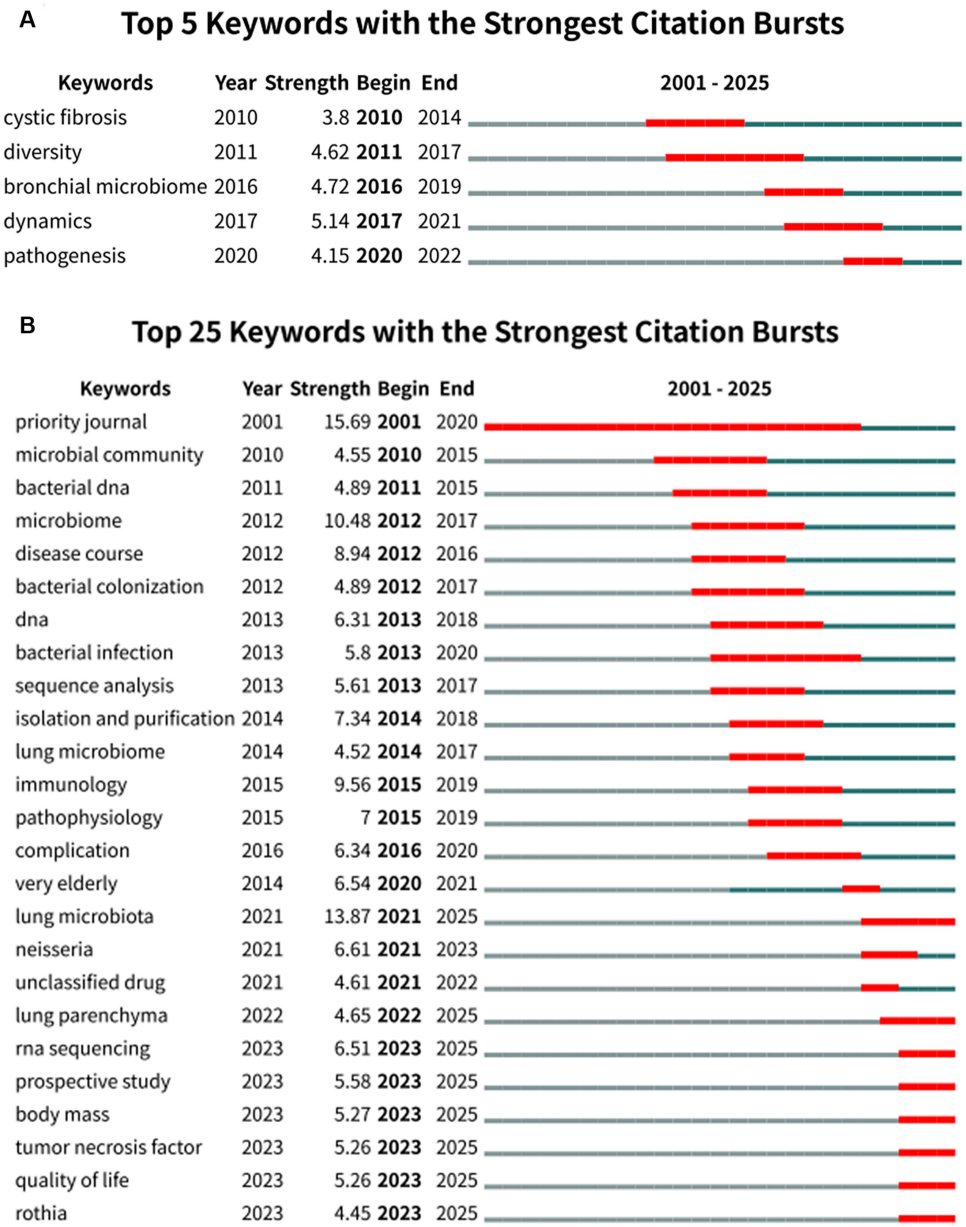
Top burst keywords identified using CiteSpace analysis in COPD and respiratory microbiota research. **(A)** Keyword citation bursts detected in the WoSCC dataset. **(B)** Keyword citation bursts detected in the Scopus dataset.

CiteSpace analysis of burst keywords revealed both shared and database-specific research emphases across the WoSCC and Scopus datasets (Figure 5). In the WoSCC dataset (Figure 5A), the strongest citation bursts were observed for dynamics (strength = 5.14), and diversity (strength = 4.62), whereas in the Scopus dataset (Figure 5B), the most prominent burst keywords included priority journal (strength = 15.69), lung microbiota (strength = 13.87), immunology (strength = 9.56), and disease course (strength = 8.94), collectively reflecting evolving research attention toward temporal microbial changes, respiratory-specific microbiota, host immune mechanisms, and disease progression in COPD.

**Figure 6:**
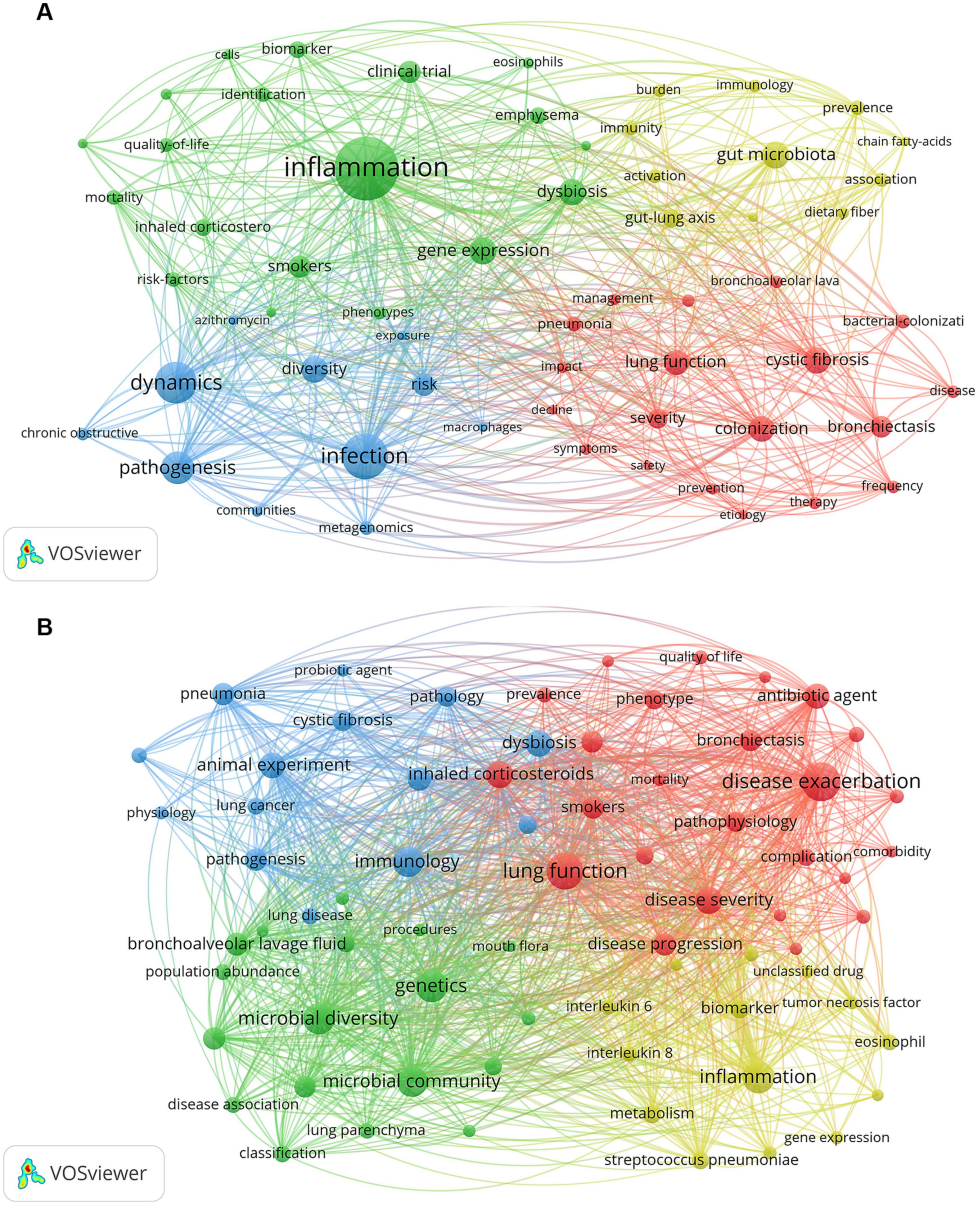
Thematic evolution of research topics in COPD and respiratory microbiota studies. **(A)** Thematic evolution map based on the WoSCC database. **(B)** Thematic evolution map based on the Scopus database.

To explore temporal changes in research themes, a thematic evolution analysis was conducted using the bibliometrix package in R. Results from the WoSCC database are shown in Figure 6A, Supplementary Table S4.

In the WoSCC dataset, research themes exhibited a clear temporal evolution. During the early stage (2013–2016), dominant themes were primarily related to classical disease entities and infection-associated terms, including chronic bronchitis, cystic fibrosis, *Pseudomonas aeruginosa*, bacteria, and bacterial colonization.

In the intermediate stage (2017–2019), keywords such as diversity, bronchial microbiota, colonization, sputum, and infection became more prominent, indicating a shift toward microbiota-oriented research themes.

In the most recent stage (2020–2024), research themes increasingly focused on respiratory microbiota, dysbiosis, dynamics, exacerbations, lung function, and risk, reflecting a transition toward clinically relevant and outcome-related topics.

Scopus-based thematic evolution analysis (Figure 6B, Supplementary Table S4) revealed a broadly similar progression, with early emphasis on microbiological and immunological concepts, followed by increased attention to molecular and sequencing-related terms, and more recent focus on respiratory microbiota, immune markers, and clinical outcomes.

### Integrated hotspot analysis

Integrated analyses of keyword co-occurrence, citation bursts, and thematic evolution across the WoSCC and Scopus databases identified three major research hotspots in COPD and respiratory microbiota studies. The first hotspot centers on respiratory microbiota composition and dynamics, characterized by frequent keywords such as respiratory microbiota, diversity, and dynamics, reflecting sustained attention to microbial dysbiosis and temporal changes in COPD. The second hotspot focuses on pathogen colonization and inflammation-related processes, with prominent terms including bacterial colonization, infection, *Haemophilus influenzae*, *Streptococcus pneumoniae*, *Pseudomonas aeruginosa*, and inflammation, highlighting persistent interest in host–pathogen interactions. The third hotspot emphasizes clinical outcomes and translational relevance, marked by keywords related to exacerbations, lung function, hospitalization, mortality, quality of life, biomarkers, and therapeutic interventions.

## Discussion

In this bibliometric study, we systematically characterized the global research landscape, intellectual structure, and evolving thematic trends of respiratory microbiota research in COPD. By integrating WoSCC and Scopus databases and applying multiple bibliometric tools, we provide a reproducible macro-level synthesis that complements existing mechanistic and meta-analytic studies. Unlike narrative reviews or meta-analyses that summarize biological findings, our study identifies structural shifts in research priorities and knowledge production patterns.

### Growth trajectory and maturation of the research field

The sustained increase in publication output observed across both databases indicates that respiratory microbiota research in COPD has transitioned from an emerging topic into a rapidly expanding and increasingly mature research domain. Particularly after 2014, the sharp rise in annual publications coincided with the widespread adoption of high-throughput sequencing technologies and improved bioinformatic pipelines, which enabled reliable characterization of low-biomass respiratory microbial communities (17, 18).

Importantly, the observed publication growth was accompanied by diversification of research themes rather than mere volume expansion. This pattern suggests that the field is not only expanding quantitatively but also evolving conceptually, with increasing attention to dynamic, functional, and clinically relevant aspects of the respiratory microbiota.

### Core journals and intellectual foundations

Journal distribution and co-citation analyses revealed a relatively stable intellectual foundation anchored in leading respiratory and microbiology journals, with the American Journal of Respiratory and Critical Care Medicine occupying a central position in both WoSCC- and Scopus-based networks. This finding is consistent with previous bibliometric analyses showing that high-impact respiratory journals play a dominant role in disseminating foundational microbiota research in COPD (19).

Highly cited references identified in this study predominantly focused on respiratory microbiota characterization, microbial dynamics during exacerbations, and host–microbe interactions. These seminal works form the conceptual backbone of the field and continue to exert strong citation influence, indicating their lasting relevance. The dispersion of influential publications across multiple journals further reflects the interdisciplinary nature of COPD microbiota research, spanning respiratory medicine, microbiology, immunology, and systems biology (20).

### Research hotspots: from dysbiosis to clinical relevance

Keyword co-occurrence and clustering analyses consistently identified three major research hotspots. The first hotspot centers on respiratory microbiota composition and dynamics highlighting sustained interest in microbial diversity community structure and temporal instability in COPD. This aligns with accumulating evidence that respiratory microbial dysbiosis is associated with disease severity and progression (21, 22).

The second hotspot focuses on pathogen colonization and inflammation-related processes. Despite the paradigm shift toward microbiota-wide analyses, pathogen-oriented keywords such as *Haemophilus influenzae*, *Pseudomonas aeruginosa*, infection, and colonization remained highly prominent. This suggests that classical pathogen-focused research continues to coexist with broader microbiota approaches, particularly in studies addressing exacerbations and inflammatory endotypes of COPD (23).

The third hotspot emphasizes clinically relevant outcomes and translational implications. Keywords related to exacerbations, lung function, hospitalization, mortality, and quality of life increasingly co-occurred with microbiota-related terms, indicating a growing effort to link microbial characteristics with patient-centered outcomes. This trend reflects an important shift toward clinical applicability and risk stratification in COPD microbiota research.

Based on the integrated hotspot analysis, future research should prioritize longitudinal cohort studies, multi-omics integration, mechanistic validation of host–microbiota interactions, and standardized sequencing methodologies.

### Emerging trends: host–microbiota interactions and integrative approaches

Citation burst detection and thematic evolution analyses further illustrated a temporal shift toward integrative and function-oriented research themes. Recent burst keywords and references increasingly involved immune regulation, microbial metabolites, and multi-omics integration. These findings are consistent with emerging studies demonstrating that respiratory microbial communities interact closely with host immune pathways and metabolic networks, influencing COPD phenotypes and disease trajectories (24, 25).

Notably, the growing prominence of multi-omics approaches reflects an effort to move beyond descriptive taxonomy toward functional interpretation of microbial ecosystems. This evolution suggests that future research will increasingly focus on causal inference and mechanistic insights, potentially facilitating the identification of novel therapeutic targets (26).

### Expansion toward systemic perspectives: the gut–lung axis

Another notable emerging theme identified in this bibliometric analysis is the gut–lung axis. Keywords related to gut microbiota, metabolism, and immune modulation exhibited increasing frequency and citation bursts in recent years. This trend mirrors a growing body of experimental and clinical evidence suggesting that gut microbial dysbiosis may influence pulmonary immune responses and COPD outcomes through systemic signaling pathways (27, 28).

The emergence of this theme highlights a conceptual expansion of COPD microbiota research from a lung-centered perspective to a systemic framework, positioning COPD as a multi-organ disorder influenced by interconnected microbial ecosystems.

## Limitations

Several limitations of this bibliometric study should be acknowledged. First, although both WoSCC and Scopus were included to enhance coverage, publications indexed exclusively in other databases may have been omitted, potentially resulting in incomplete literature representation. Second, citation-based indicators are inherently influenced by publication age and database indexing practices, which may favor older or highly visible studies. Third, keyword co-occurrence and thematic evolution analyses rely on author-provided keywords and database indexing terms, which may not fully capture emerging concepts or methodological nuances. Moreover, bibliometric analysis reflects research activity and thematic emphasis rather than providing direct biological or clinical evidence. Consequently, the findings should be interpreted as indicators of research evolution rather than definitive conclusions regarding disease mechanisms or therapeutic efficacy. Finally, the present bibliometric analysis did not account for potential influences of comorbid conditions such as tuberculosis and HIV, nor did it capture regional characteristics of underrepresented areas, particularly in Africa, where such coinfections may considerably shape the composition of the respiratory and gut microbiota and modulate the pathophysiology and outcomes of COPD. Future studies, ideally through international collaborative efforts, should prioritize these underrepresented settings to address this gap and provide a more globally representative understanding of the microbiota–COPD interplay.

Despite these limitations, this study offers a comprehensive and objective framework for understanding the evolution of respiratory microbiota research in COPD. The increasing focus on microbial dynamics, host–microbiota interactions, and clinically relevant outcomes suggests that future investigations are likely to prioritize longitudinal designs, standardized methodologies, and integrative multi-omics strategies. Strengthening international collaboration and expanding research efforts in underrepresented regions may further enhance the generalizability and translational impact of this field.

## Data Availability

The original contributions presented in the study are included in the article/Supplementary material, further inquiries can be directed to the corresponding author.
