# Supplementary material for: Global research trends and thematic evolution of respiratory microbiota in COPD: a bibliometric study
Source: Front Med (Lausanne). 2026 Apr 9;13:1780141. doi: 10.3389/fmed.2026.1780141 (PMC13102642; doi:10.3389/fmed.2026.1780141)
Supplement: Supplementary file 1 [file Data_sheet_1.zip › Supplementary Material/Search strategy.docx]

Search Strategy

The WoSCC search strategy was as follows: TS = ((COPD OR "chronic obstructive pulmonary disease") AND ("airway microbiome" OR "lung microbiome" OR "respiratory microbiome" OR "airway microbiota" OR "lung microbiota" OR "respiratory microbiota" OR "airway microorganism" OR "lung microorganism" OR "respiratory microorganism" OR "airway microbe" OR "lung microbe" OR "respiratory microbe" OR "airway germ" OR "lung germ" OR "respiratory germ" OR "airway microbiology" OR "lung microbiology" OR "respiratory microbiology" OR "alveolar microbiome" OR "alveolar microbiota" OR "alveolar microorganism" OR "alveolar microbe" OR "alveolar germ" OR "alveolar microbiology" OR "sputum microbiome" OR "sputum microbiota" OR "sputum microorganism" OR "sputum microbe" OR "sputum germ" OR "sputum microbiology" OR "pulmonary microbiome" OR "pulmonary microbiota" OR "pulmonary microorganism" OR "pulmonary microbe" OR "pulmonary germ" OR "pulmonary microbiology" OR "lung tissue microbiome" OR "lung tissue microbiota" OR "lung tissue microorganism" OR "lung tissue microbe" OR "lung tissue germ" OR "lung tissue microbiology"OR "bronchial microbiome" OR "bronchial microbiota" OR "bronchial microorganism" OR "bronchial microbe" OR "bronchial germ" OR "bronchial microbiology" OR "bronchic microbiome" OR "bronchic microbiota" OR "bronchic microorganism" OR "bronchic microbe" OR "bronchic germ" OR "bronchic microbiology")) AND LA = (English) AND DT = (Article OR Review) AND DOP = (2000-01-01/2025-12-18).

The Scopus search strategy was: TITLE-ABS-KEY ( ( COPD OR "chronic obstructive pulmonary disease" ) AND ( "airway microbiome" OR "lung microbiome" OR "respiratory microbiome" OR "airway microbiota" OR "lung microbiota" OR "respiratory microbiota" OR "airway microorganism" OR "lung microorganism" OR "respiratory microorganism" OR "airway microbe" OR "lung microbe" OR "respiratory microbe" OR "airway germ" OR "lung germ" OR "respiratory germ" OR "airway microbiology" OR "lung microbiology" OR "respiratory microbiology" OR "alveolar microbiome" OR "alveolar microbiota" OR "alveolar microorganism" OR "alveolar microbe" OR "alveolar germ" OR "alveolar microbiology" OR "sputum microbiome" OR "sputum microbiota" OR "sputum microorganism" OR "sputum microbe" OR "sputum germ" OR "sputum microbiology" OR "pulmonary microbiome" OR "pulmonary microbiota" OR "pulmonary microorganism" OR "pulmonary microbe" OR "pulmonary germ" OR "pulmonary microbiology" OR "lung tissue microbiome" OR "lung tissue microbiota" OR "lung tissue microorganism" OR "lung tissue microbe" OR "lung tissue germ" OR "lung tissue microbiology" OR "bronchial microbiome" OR "bronchial microbiota" OR "bronchial microorganism" OR "bronchial microbe" OR "bronchial germ" OR "bronchial microbiology" OR "bronchic microbiome" OR "bronchic microbiota" OR "bronchic microorganism" OR "bronchic microbe" OR "bronchic germ" OR "bronchic microbiology" ) ) AND PUBYEAR > 2000 AND PUBYEAR < 2026 AND ( LIMIT-TO ( DOCTYPE , "ar" ) OR LIMIT-TO ( DOCTYPE , "re" ) ) AND ( LIMIT-TO ( LANGUAGE , "English" ) ).
