# Supplementary figures and images for: Global research trends and thematic evolution of respiratory microbiota in COPD: a bibliometric study
Source: Front Med (Lausanne). 2026 Apr 9;13:1780141. doi: 10.3389/fmed.2026.1780141 (PMC13102642; doi:10.3389/fmed.2026.1780141)

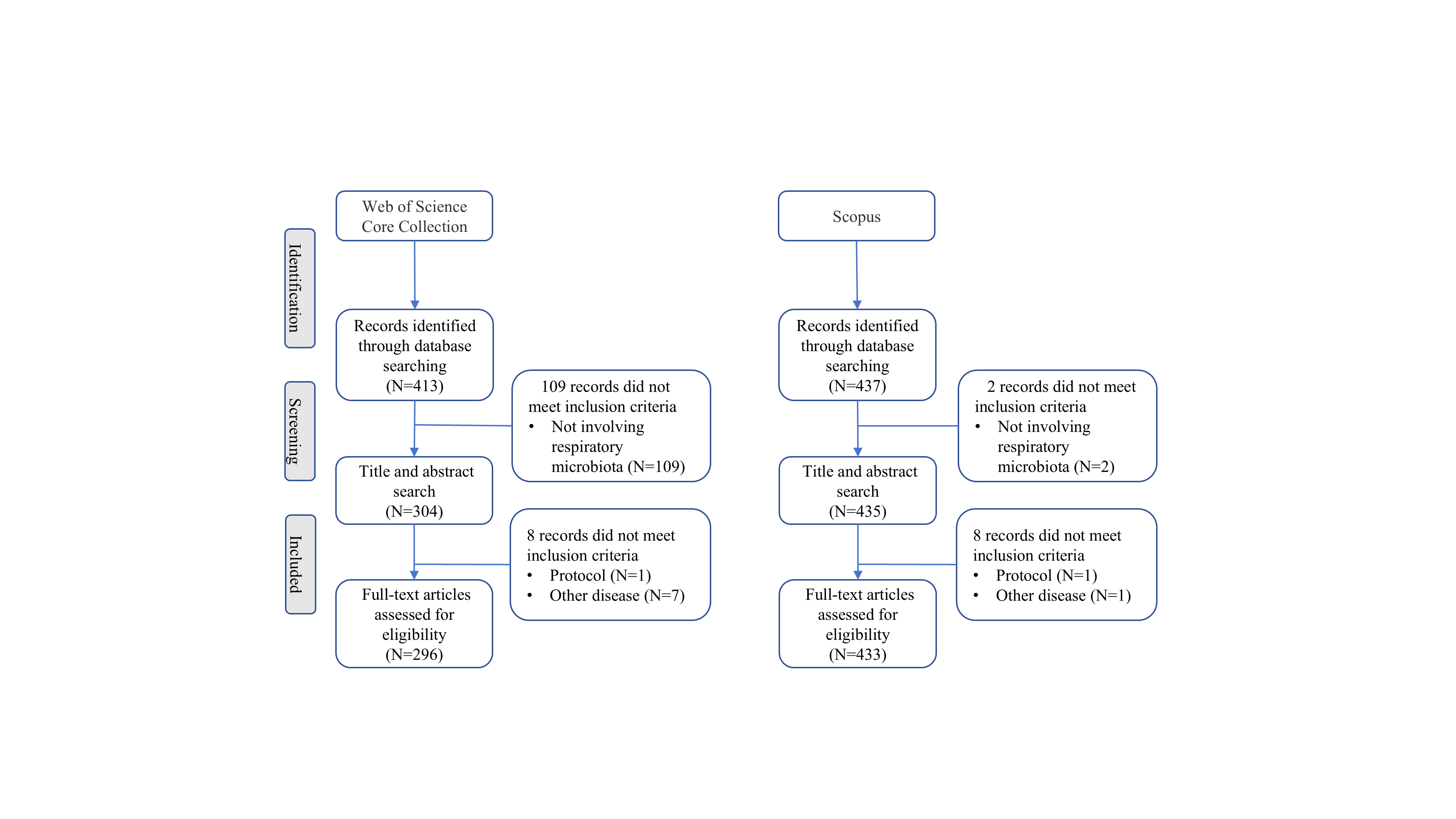

Supplement: Supplementary file 1 [file Data_sheet_1.zip › Supplementary Material/Supplementary Figure S1.tif]
